# Learning curve for full-endoscopic lumbar decompression via interlaminar approach using the learning curve cumulative summation analysis

**DOI:** 10.1186/s13018-025-05699-y

**Published:** 2025-03-19

**Authors:** Sung Cheol Park, Sang-Min Park, Hoon-Jae Chung, Yong Jin You

**Affiliations:** 1 Spine Endoscopy Center, Department of Orthopaedic Surgery, Bumin Hospital Seoul, Seoul, 07590 South Korea; 2 Spine Center, Department of Orthopaedic Surgery, Seoul National University College of Medicine, Seoul National University Bundang Hospital, Seongnam, 13620 Gyeonggi-do South Korea

**Keywords:** Learning curve, Full-endoscopic spine surgery, Lumbar spinal stenosis, Interlaminar approach

## Abstract

**Background:**

Full-endoscopic spine surgery (FESS) is generally considered to have a steep learning curve due to its technical complexity. This study aimed to evaluate the learning curve for full-endoscopic decompressive laminectomy via the interlaminar approach using learning curve cumulative summation test (LC-CUSUM) analysis, which provides objective statistical monitoring of surgical competency acquisition, and determine the number of cases required for surgical competency.

**Methods:**

We retrospectively analyzed the first 60 consecutive patients who underwent single-level interlaminar endoscopic unilateral lumbar decompression for lumbar spinal stenosis performed by a single surgeon with 4 years of experience. LC-CUSUM analysis was employed with operative time as the primary outcome measure. The target time was set at 80 min, based on the same surgeon’s mean operative time for microscopic laminectomy. The patients were divided into the early (≤ 30 cases) and late (> 30 cases) learning periods and compared.

**Results:**

LC-CUSUM analysis revealed that competency was achieved after 51 cases. The mean operative time significantly decreased from 90.20 ± 24.44 min in the early period to 71.47 ± 16.65 min in the late period (*p* = 0.001). Estimated blood loss showed significant reduction (54.83 ± 42.58 ml vs. 34.83 ± 19.10 ml, *p* = 0.024). Complication rates remained consistent between periods (10% each), with similar rates of dural tears (6.67% in both periods).

**Conclusions:**

The results of this study have demonstrated that a learning period of 51 cases could be required to achieve proficiency in full-endoscopic interlaminar lumbar decompression. However, the procedure can be safely performed even during the early learning period by surgeons with adequate microscopic surgical experience.

## Introduction

As society ages, the number of patients with degenerative spinal diseases who have various medical comorbidities has been increasing [1]. Consequently, spine surgeons increasingly encounter patients with significant frailty in their clinical practice. This trend has heightened interest in minimally invasive spinal surgery to reduce surgical burden, leading to the rapid evolution and growing popularity of endoscopic spine surgery (ESS) [2, 3]. ESS can reduce damage to anatomical structures such as muscles, ligaments, and bone, resulting in less postoperative pain, faster recovery, and a reduced length of hospital stay (LOS) [4, 5]. However, it remains challenging even for experienced spine surgeons. In particular, full-endoscopic spine surgery (FESS), a variant of ESS, is generally considered to have a steep learning curve due to its technical complexity [6].

In its early stages, FESS was limited to transforaminal soft tissue procedures because of limitations in surgical exposure and instrumentation [1, 7, 8]. However, ongoing advancements in surgical techniques and equipment have overcome these technical constraints and broadened the scope of FESS treatments [2, 8]. Advances in endoscopic instruments, including larger working channels, endoscopic drills, and specialized forceps, coupled with evolving approaches and surgical techniques, have made decompressive surgery for spinal stenosis feasible [1, 9]. Moreover, innovations in FESS are still ongoing, as evidenced by recent reports of endoscopic-assisted interbody fusion [10, 11].

The learning curve in spine surgery may be associated with postoperative outcomes and the risk of various critical complications. Given that ESS often requires considerable experience and training to achieve proficiency, numerous studies have examined its learning curve [6, 12–16]. Among various analytical methods, learning curve cumulative summation (LC-CUSUM) analysis is designed to monitor procedural progress over time and evaluate achievement of defined proficiency levels [6, 16, 17]. Additionally, it offers visual representation that facilitates data interpretation. Although several studies have examined the learning curve for FESS [12, 13, 15, 18, 19], to our knowledge, no study has evaluated the learning curve using LC-CUSUM for full-endoscopic decompressive surgery via the interlaminar approach in lumbar spinal stenosis. Therefore, this study aims to evaluate the learning curve of interlaminar endoscopic unilateral lumbar decompression (IE-ULD) using full-endoscopy and to suggest the number of cases required for competency using LC-CUSUM analysis of a single surgeon’s experience.

## Materials and methods

### Study patients

The current retrospective study was approved by the institutional review board of our institution, and a waiver of consent was obtained (BMH 2024-12-022). We reviewed the medical records of consecutive patients who underwent single-level IE-ULD using full-endoscopy for central canal or lateral recess stenosis in the lumbar spine from the surgeon’s first case onward. All procedures were performed by a single orthopaedic spine surgeon with 4 years of experience (first author) from August 2023 through June 2024. Patients with unilateral neurogenic intermittent claudication and/or radiculopathy refractory to conservative treatments for at least 6 weeks, or those with neurologic deficits, were included. Patients with infection, history of previous lumbar surgery at the same level, or incomplete medical record documentation were excluded.

Accordingly, a total of 64 patients were identified. However, patients with infection (*n* = 1), history of previous lumbar surgery at the same level (*n* = 2), or incomplete documentation (*n* = 1) were excluded, leaving 60 patients.

### Surgical technique

The patient was placed prone on a Wilson frame under general anesthesia. The surgical level was confirmed under C-arm fluoroscopy guidance. After making a 9 mm skin incision targeting the caudal border of the upper lamina, a dilator was inserted for blunt dissection. The working sleeve was then inserted over the dilator with its beveled opening oriented medially. A 20°/9.3 × 7.4 mm VERTEBRIS stenosis Endoscopic System (RIWOspine GmbH, Knittlingen, Germany) was introduced through the working sleeve. The procedure was performed under constant saline irrigation.

**Fig. 1:**
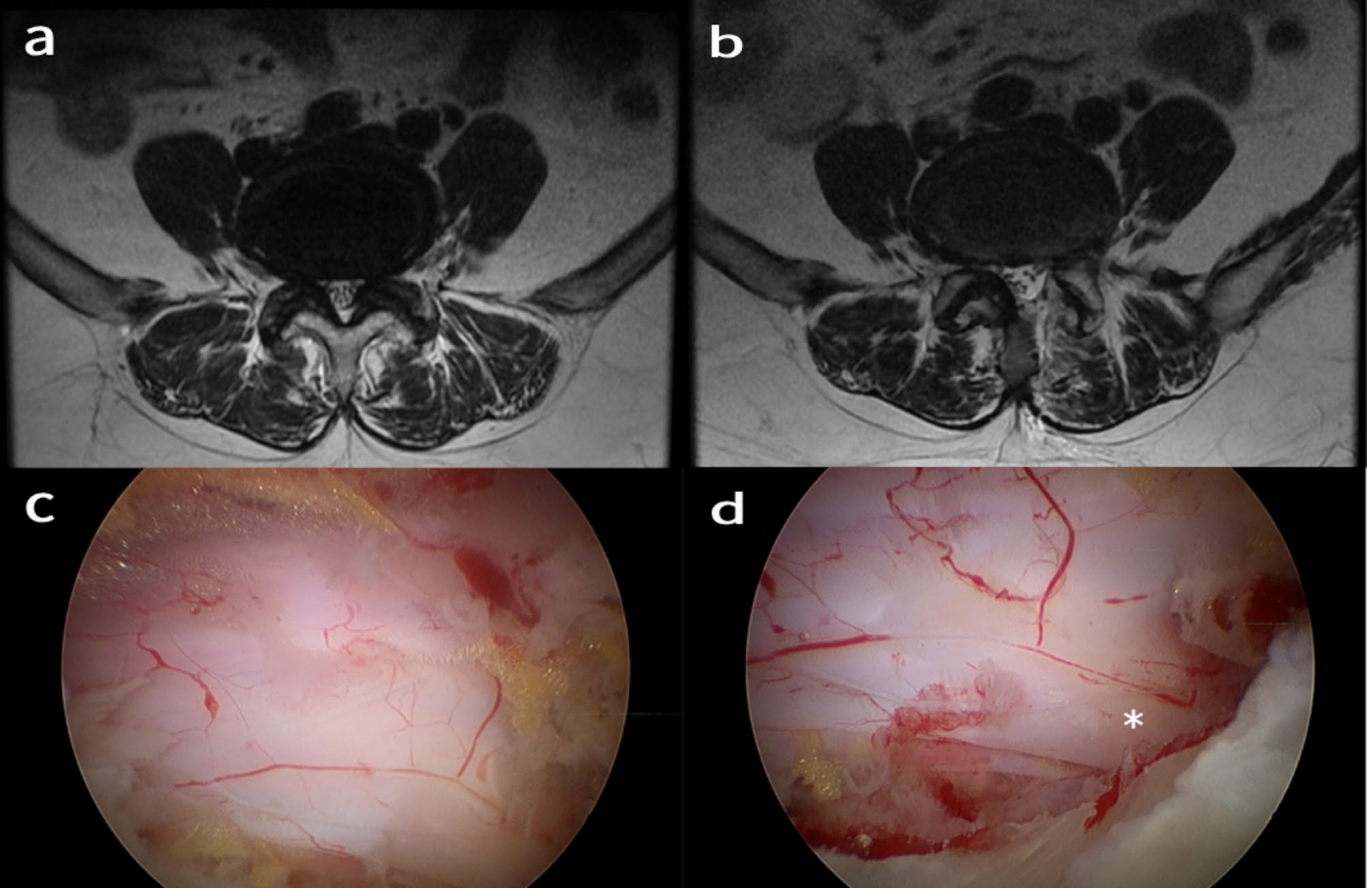
Full-endoscopic unilateral lumbar decompression via interlaminar approach in a 66-year-old female patient. **(a)** Preoperative MRI demonstrating central canal stenosis at the L4-5 level. **(b)** Postoperative MRI revealing complete decompression of the dural sac on the left side. **(c, d)** Intraoperative endoscopic views showing the decompressed dural sac and ipsilateral nerve root (asterisk)

The inferior margin of the upper lamina was exposed using an electrocautery probe and micro-rongeur. Subsequently, we performed ipsilateral decompression with cranial and caudal laminotomy using an endoscopic high-speed drill and Kerrison punch. Medial facetectomy was carried out to achieve bony decompression extending to the medial margin of the lower pedicle laterally. Following bony work, the ligamentum flavum was removed en bloc. Successful decompression was verified by restoration of dural pulsation. Hemostasis was achieved using bipolar radiofrequency cautery and hemostatic agents (Fig. 1).

### Data collection

Medical records were reviewed to collect information on demographics (age, sex, body mass index), preoperative stenosis grade, operative details (operating time, operative level, operative side, estimated blood loss [EBL]), LOS, perioperative complications, and clinical outcomes (visual analogue scale [VAS], Oswestry Disability Index [ODI]). The operating time was defined as the time from skin incision to wound closure in this study.

### Statistical analysis

The LC-CUSUM represents a modification of traditional CUSUM analysis specifically designed to monitor the acquisition of surgical competency. This method evaluates the transition from an ‘out-of-control’ state during initial learning to an ‘in-control’ state once proficiency is achieved. Unlike conventional CUSUM testing, LC-CUSUM inverts the null hypothesis (H0), where H0 represents inadequate performance and H1 represents adequate performance.

For our analysis, operative duration served as the primary success criterion, measured from initial incision to wound closure according to anesthesia records. The target time was established at 80 min, based on the mean operative duration for microscopic laminectomy performed by the same surgeon. Cases exceeding this threshold were classified as procedural failures. The statistical parameters were established through departmental expert consensus, with acceptable performance threshold (p1) set at 20% and unacceptable performance threshold (p0) at 40%. Type I error (α) was set at 0.05 and Type II error (β) at 0.20.

These parameters yielded a success credit (S) of -0.2933 units and a failure penalty (1-S) of + 0.7067 units, with a decision limit (h) of -2.8268. The resulting analysis displays competency acquisition through downward trending slopes for successful procedures and upward trends for failures. Competency is achieved when the cumulative score crosses below the decision limit h. A holding barrier at zero prevents excessive negative accumulation from multiple early failures.

Upon achieving initial competency via LC-CUSUM, standard CUSUM monitoring was implemented with identical parameters to assess sustained performance, using a recalculated decision interval h of 2.8268. This analytical framework provides a robust method for evaluating both the acquisition and maintenance of surgical proficiency through objective performance metrics.

For calculating LC-CUSUM and CUSUM scores, we used Excel software (Excel 2020, Microsoft, Redmond, WA). Other statistical analyses were performed using SPSS version 25.0 (IBM Corp., Armonk, NY, USA). Continuous variables were expressed as means ± standard deviations, and categorical variables were expressed as frequencies and percentages. Comparisons between the early (≤ 30 cases) and late (> 30 cases) learning periods were performed using Student’s t-test for continuous variables and the chi-square test or Fisher’s exact test for categorical variables. Comparison of clinical outcomes over time between the two groups was analyzed using repeated measures analysis of variance (RM-ANOVA). Paired t-tests were used to compare preoperative and 6-month postoperative clinical outcomes within each group. A p-value < 0.05 was considered statistically significant.

## Results

The baseline characteristics between early and late groups showed no significant differences in age (55.70 ± 15.91 vs. 61.77 ± 11.71 years, *p* = 0.098), sex distribution (female: 46.7% vs. 50.0%, *p* = 0.796), and operative side (right: 36.7% vs. 33.3%, *p* = 0.787) (Table 1).

**Table 1:** Comparison of baseline characteristics between patients in early and late learning period

	Early period (*n* = 30)	Late period (*n* = 30)	*p*-value
Age (years)	55.70 ± 15.91	61.77 ± 11.71	0.098
Sex, *n* (%)			0.796
Female	14 (46.7)	15 (50.0)	
Male	16 (53.3)	15 (50.0)	
Operative side, *n* (%)			0.787
Right	11 (36.7)	10 (33.3)	
Left	19 (63.3)	20 (66.7)	
Operative level, *n* (%)			0.186
L1-2	0 (0)	1 (3.3)	
L2-3	1 (3.3)	1 (3.3)	
L3-4	4 (13.3)	10 (33.3)	
L4-5	14 (46.7)	13 (43.3)	
L5-S1	11 (36.7)	5 (16.7)	

The operative outcomes demonstrated significant improvement in the late period compared to the early period. The mean operative time decreased significantly from 90.20 ± 24.44 min in the early period to 71.47 ± 16.65 min in the late period (*p* = 0.001). The EBL also showed significant reduction (54.83 ± 42.58 ml vs. 34.83 ± 19.10 ml, *p* = 0.024).

**Fig. 2:**
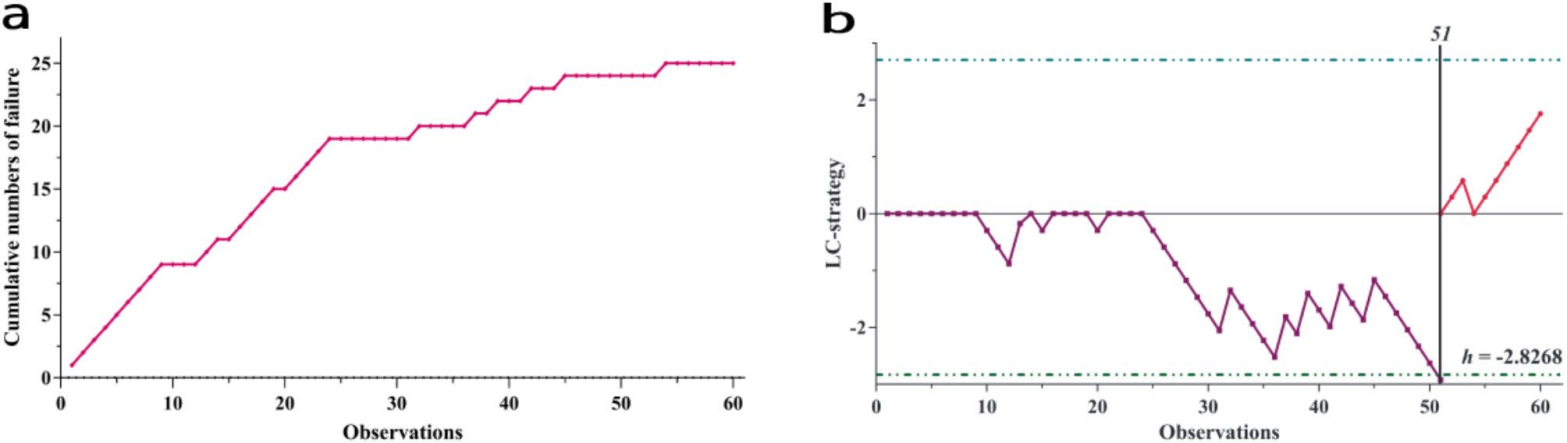
**(a)** Cumulative number of surgical failures. **(b)** Learning curve cumulative summation analysis demonstrating surgical competency achieved after the 51st operation

The LC-CUSUM analysis demonstrated that the learning curve was achieved at the 51st case, where the score crossed the predefined decision limit. This indicates that after 51 cases, the surgeon achieved consistent operative times, which was considered as proficiency criterion. The success rate for achieving target operative time substantially improved after the learning curve was reached (Fig. 2).

**Fig. 3:**
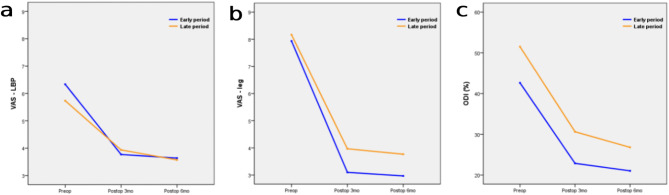
Comparison of clinical outcomes between early and late learning periods. **(a)** VAS-LBP. **(b)** VAS-leg. **(c)** ODI. VAS, visual analogue scale; LBP, low back pain; ODI, Oswestry Disability Index

The overall complication rate was comparable between early and late periods (10% each). Dural tear occurred in 2 cases (6.67%) in both periods. One case of root injury (3.33%) occurred in the late period, while one case of incomplete decompression (3.33%) was noted in the early period. No postoperative hematoma was observed in either group (Table 2). RM-ANOVA showed no significant differences in VAS and ODI between the two groups (*p* > 0.05) (Fig. 3). Based on paired t-tests, both early and late periods showed significantly improved VAS and ODI at 6 months postoperatively compared to preoperative status (*p* < 0.001) (Table 3).

**Table 2:** Comparison of perioperative outcomes between patients in early and late learning period

	Early period (*n* = 30)	Late period (*n* = 30)	*p*-value
Operating time (min)	90.20 ± 24.44	71.47 ± 16.65	0.001^*^
Estimated blood loss (ml)	54.83 ± 42.58	34.83 ± 19.10	0.024^*^
Length of hospital stay (days)	4.80 ± 4.28	5.83 ± 2.64	0.265
Complications, *n* (%)	3 (10)	3 (10)	1.000
Dural tear	2 (6.67)	2 (6.67)	
Root injury	0 (0)	1 (3.33)	
Incomplete decompression	1 (3.33)1	0 (0)	
Hematoma	0 (0)	0 (0)	

^*^Statistically significant (*p* < 0.05)

**Table 3:** Comparison of clinical outcomes between preoperative and postoperative 6-month status

	Preoperative	Postoperative 6-month	*p*-value	
**Early learning period**				
VAS– LBP	6.33 ± 2.04	3.63 ± 2.13	< 0.001^*^	
VAS– leg	7.93 ± 2.64	2.97 ± 2.20	< 0.001^*^	
ODI (%)	42.63 ± 20.96	21.03 ± 14.81	< 0.001^*^	
**Late learning period**				
VAS– LBP	5.73 ± 1.60	3.57 ± 1.91	< 0.001^*^	
VAS– leg	8.17 ± 1.34	3.77 ± 1.79	< 0.001^*^	
ODI (%)	51.51 ± 18.50	26.80 ± 15.70	< 0.001^*^	

Abbreviations: VAS, visual analogue scale; LBP, low back pain; ODI, Oswestry Disability Index

^*^ Statistically significant (*p* < 0.05)

## Discussion

We evaluated the level of difficulty of full-endoscopic lumbar decompression surgery via interlaminar approach by assessing the learning curve using LC-CUSUM analysis. Our results indicated that a considerable learning period, 51 operations, might be required to reach adequate proficiency as the level of the same procedure using the microscope. However, no significant difference was found in perioperative complications and clinical outcomes between the early and late learning period, suggesting that this surgery can be performed with caution even during the learning period.

Although FESS has been traditionally considered to have a steep learning curve [6, 20], our findings suggest that the learning period of FESS may not be significantly different from that of biportal ESS (BESS). A previous study on BESS reported that 58 cases were required to achieve proficiency, which is comparable to our finding of 51 cases for FESS [6]. However, direct comparison between these studies should be made with caution due to methodological differences; while the previous BESS study used a senior surgeon’s microscopic surgery outcomes as their success criteria, our study used the same surgeon’s microscopic surgery outcomes as the reference. Nevertheless, the similarity in the number of cases required for competency between FESS and BESS is noteworthy, as it challenges the conventional perception of FESS having a particularly steep learning curve. This finding may help reduce psychological barriers for surgeons considering the adoption of FESS in their practice.

While BESS has the advantage of utilizing conventional surgical instruments, enabling familiar procedural execution for spine surgeons, recent advances in FESS instrumentation have significantly expanded its surgical indications [8]. Modern FESS equipment now allows for procedures comparable to microscopic surgery. Unlike BESS, which requires different portal positions based on the approach side [11, 21], FESS maintains consistent portal positioning regardless of the approach side, potentially reducing anatomical confusion during endoscopic visualization. This technical consistency was reflected in our operative outcomes, with no significant differences in operative times between right-sided (*n* = 21) and left-sided (*n* = 39) approaches (80.57 ± 20.70 vs. 80.97 ± 24.09 min, *p* = 0.949) during the learning period. Additionally, FESS may offer superior minimization of muscle injury compared to BESS, as it requires only a single small working portal, resulting in smaller working space over the muscle-laminar interface [22–24]. The advancement in endoscopic equipment combined with these technical advantages suggests that FESS can be a viable alternative to both conventional microscopic surgery and BESS for appropriate surgical candidates.

The learning curve in surgical procedures can be evaluated using various parameters, including clinical outcomes, operative time, LOS, complication rates, and EBL [13, 14]. Among these, we utilized operative time as the primary indicator in our LC-CUSUM analysis, as LC-CUSUM is a particularly useful statistical tool for assessing whether a surgeon has reached an adequate level of competency. While previous studies using LC-CUSUM analysis often set their target operative times based on other surgeons’ mean operative times [6, 16, 19], we believe this approach may have limitations due to potential variations in surgical techniques and endpoints among different surgeons. Therefore, in this study, we used the mean operative time of conventional microscopic surgery performed by the same surgeon as our reference point. This methodology of using a single surgeon’s conventional surgical times as the competency goal provides a more standardized and relevant benchmark, as it eliminates the confounding effects of inter-surgeon variability in surgical technique, decision-making, and surgical endpoints. This approach may offer a more accurate assessment of the learning curve by directly comparing different surgical techniques within the same operator’s experience.

Interestingly, while there was a significant difference in operative times between the early and late learning periods (90.20 ± 24.44 vs. 71.47 ± 16.65 min, *p* = 0.001), the complication rates remained similar (10% in both groups). This finding can be attributed to several factors. First, although the technical proficiency in handling full-endoscopic instruments required time to develop, the surgeon’s extensive experience in microscopic decompressive laminectomy likely provided a solid foundation in surgical anatomy and decompression techniques. Second, the magnified endoscopic visualization offered clear anatomical identification, potentially helping to prevent complications even during the early learning period. The superior visualization may have compensated for the initial technical unfamiliarity with endoscopic instruments. Additionally, the surgeon’s prior experience with BESS might have contributed to the low complication rate during the early learning period, although this potential influence requires further investigation in future studies. These findings suggest that experienced spine surgeons can safely transition to FESS, maintaining acceptable complication rates even during their learning phase.

This study has several limitations that should be acknowledged. First, as a retrospective study, it is inherently subject to selection bias and potential confounding factors. Second, while we used total operative time as the criterion for determining success or failure, operative time can be influenced by various factors beyond surgical technique, including the extent of epidural bleeding, bone density, severity of stenosis, and the degree of inflammation in the epidural space. Third, the surgeon’s prior experience with BESS may have created a favorable bias, as familiarity with endoscopic visualization and basic endoscopic concepts might have shortened the learning curve. Fourth, this study focused on single-level decompressions only, and the learning curve might differ for multi-level or more complex procedures. Fifth, our follow-up period was relatively short, and long-term outcomes and complications were not evaluated. Despite these limitations, to the best of our knowledge, this is the first study to evaluate the learning curve for FESS based on a single surgeon’s experience using LC-CUSUM analysis, providing valuable insights into the transition from microscopic to full-endoscopic techniques.

## Conclusion

This LC-CUSUM analysis demonstrated that approximately 51 cases were required to achieve competency in IE-ULD for spinal stenosis. While this indicates a substantial learning period, both the complication rates and clinical outcomes remained consistently comparable even during the early learning phase, suggesting that FESS can be safely implemented by surgeons with sufficient microscopic surgical experience. These findings suggest that FESS represents a feasible alternative to conventional microscopic surgery, with an acceptable learning curve comparable to that of BESS.

## Data Availability

The datasets used and/or analysed during the current study are available from the corresponding author on reasonable request.

## Abbreviations

ESS: Endoscopic spine surgery
LOS: Length of hospital stay
FESS: Full-endoscopic spine surgery
LC-CUSUM: Learning curve cumulative summation
IE-ULD: Interlaminar endoscopic unilateral lumbar decompression
EBL: Estimated blood loss
BESS: Biportal endoscopic spine surgery
